# Depression Among Keratoconus Patients in Saudi Arabia

**DOI:** 10.7759/cureus.11932

**Published:** 2020-12-06

**Authors:** Walaa Al-Dairi, Omar M AL Sowayigh, Ali A Al Saeed, Ali Alsaad

**Affiliations:** 1 Surgery, College of Medicine, King Faisal University, Al-Ahsa, SAU; 2 Ophthalmology, College of Medicine, King Faisal University, Al-Ahsa, SAU; 3 Psychiatry, College of Medicine, King Faisal University, Al-Ahsa, SAU

**Keywords:** depression, keratoconus, 9-item patient health questionnaire (phq-9), corrective contact lens

## Abstract

Background

Depression is a highly prevalent disorder globally and locally in Saudi Arabia. Individuals with chronic conditions are more liable to develop depression. Keratoconus is a chronic progressive corneal disorder that markedly affects the vision and quality of life, making its sufferers liable to developing depression.

Methods

This is a descriptive cross-sectional study that was conducted using 9-item Patient Health Questionnaire (PHQ-9) to screen for depression among adults aged between 18 and 60 years old only. The participants in this study are patients who have been previously diagnosed with keratoconus by their ophthalmologists. The structured questionnaire was distributed using Google Forms through various social media platforms. After extracting the data, it was revised, coded and then analyzed using the Statistical Packages for Social Sciences (SPSS), version 21 (IBM Corp., Armonk, NY).

Results

A total of 330 keratoconus patients living in Saudi Arabia were recruited in this study. The modal age group was 31-40 years old (44.5%), and the male to female ratio was 3:2. The most frequently reported concurrent eye diseases of the patients were astigmatism (48.5%) and myopia (36.7%). The prevalence of depression among patients with keratoconus was 40.6% (n = 134). The use of corrective contact lens (includes both: hybrid and rigid lens) in both eyes contributed to a significantly higher depression rate among its wearers compared to users in one eye and non-users (p<0.001).

Conclusion

Depression is highly prevalent among keratoconus patients. This is especially true among corrective contact lens wearers of both eyes. Keratoconus is associated with depression regardless of disease severity and socio-demographic characteristics.

## Introduction

Keratoconus is a chronic progressive ectatic corneal disorder that is bilateral but usually develops asymmetrically [1]. It is characterized by developing a central cone-shaped bulging of the cornea that is explained by its underlying pathophysiology of stromal thinning [2]. The disease starts to develop relatively early in life, becoming especially apparent and symptomatic during puberty [3,4], progressing further and does not stabilize until the thirties of age [4]. Patients often complain of progressive deterioration of vision bilaterally which is attributed to increasing myopia and irregular astigmatism.

The prevalence of keratoconus varies widely in different geographical locations of the world, ranging from 0.3 per 100,000 in Russia to 2,300 per 100,000 in Central India [5,6]. Both genders are affected by keratoconus, however, the literature is divided between which gender is affected more; some showing males to have a higher prevalence [7] and others finding females to be higher [2] while others found no significant differences between the two [3]. In Saudi Arabia, it is more prevalent and found to be more advanced at the time of diagnosis compared to other regions of the world with colder climates and less UV light exposure [8,9]. These factors along with eye rubbing & atopy are well-recognized risk factors, nevertheless, it is believed that the predisposing cause behind it is multifactorial.

Depression is a common, frequently undiagnosed, and serious mental health disorder that is potentially life-threatening. Along with other mental health disorders, depression poses a great burden on public health worldwide [10]. It is associated with increased disability, morbidity, mortality and suicidal attempts [10,11]. It is more likely to affect the elderly and patients with chronic health conditions [12,13]. Additionally, it was found to be more common among females [11], including females in Saudi Arabia [14].

Screening for depression using questionnaires and self-rating scales has been helpful in the primary care settings [15,16]. Its use also extends to research purposes in objectively evaluating the possible presence of depression among different studied populations [15,16]. 9-item Patient Health Questionnaire (PHQ-9) and Zung Depression Inventory-Self-Rating Depression Scale (Zung SDS) questionnaires have been used in evaluating depression among keratoconus patients and were found to be both consistent and sensitive [17].

Vision is an extremely valued sense that affects every single detail of our daily life activities, and hence marked deterioration in visual function is related to impairment in one’s psychological condition and quality of life [18,19], and therefore it has been linked to depression [5,17]. Even though some studies did not demonstrate a relationship between keratoconus and depression [20,21], there is on the other hand supporting evidence that the direct relationship does exist [5,17]. By using PHQ-9, we aim in this study to estimate the prevalence of depression among keratoconus patients and explore potential factors implicated in the development of depression among keratoconus patients in Saudi Arabia.

## Materials and methods

Study design and participants

This is a descriptive cross-sectional study that was conducted using PHQ-9 questionnaire to screen for depression. The structured questionnaire was distributed using Google Forms through various social media platforms. The study included adults who were previously diagnosed with keratoconus. All individuals who were younger than 18 or older than 60 were excluded. The data variables that were collected were: age, sex, occupation, province/city, eye conditions, eye procedures and use of corrective eyeglasses or contact lens.

Statistical analysis

Descriptive statistics were presented using numbers, percentages, mean and standard deviation whenever appropriate. The relationship between the level of depression among the socio-demographic characteristics, other disorders and procedures are done for the patients had been conducted using Chi-square test. P-value of <0.05 was considered statistically significant. All statistical data were analyzed using the Statistical Packages for Social Sciences, version 21 (SPSS: IBM Corp., Armonk, NY).

Ethical consideration

This study was conducted upon the approval of the research ethics committee of the College of Medicine, King Faisal University. All data collected is confidential and a consent was taken from the participants.

## Results

A total of 330 keratoconus patients living in Saudi Arabia were recruited in this study. As seen in Table 1, the modal age group was 31-40 years old (44.5%). The male to female ratio was 3:2 and were mostly Saudis (88.5%). Nearly two-thirds (63.6%) were currently employed. The majority of patients were from the Central region (45.8%) and the majority were living in a city (82.4%).

**Table 1:** Socio-demographic characteristics of patients (n = 330)

Study variables	N (%)
Age group	
18 – 30 years	146 (44.2%)
31 – 40 years	147 (44.5%)
>40 years	37 (11.2%)
Gender	
Male	196 (59.4%)
Female	134 (40.6%)
Nationality	
Saudi	292 (88.5%)
Non-Saudi	38 (11.5%)
Employment status	
Unemployed	86 (26.1%)
Employed	210 (63.6%)
Student	34 (10.3%)
Residence region	
Central region	151 (45.8%)
Eastern region	34 (10.3%)
Northern region	27 (08.2%)
Southern region	43 (13.0%)
Western region	75 (22.7%)
Residence area	
City	272 (82.4%)
Village	58 (17.6%)

Table 2 shows the other eye disorders and the treatment done on the patients. It was revealed that the most frequently reported concurrent eye diseases of the patients were astigmatism (48.5%) and myopia (36.7%). We also found that 60.9% of the patients were using corrective eyeglasses. The proportion of patients who were using a corrective contact lens, who underwent corneal transplantation and post lens implantation in both eyes were 56.7%, 5.5% and 1.8%, respectively. In addition, there were 47.6% of patients who underwent other eye procedures.

**Table 2:** Patients’ other eye disorders and procedures (n = 330)

Parameters	N (%)
Other eye disorders	
Astigmatism	160 (48.5%)
Myopia	121 (36.7%)
Hyperopia	21 (06.4%)
Amblyopia	23 (07.0%)
Other	03 (01.4%)
Use of corrective eyeglasses	
Yes	201 (60.9%)
No	129 (39.1%)
Use of corrective contact lens	
Single eye	67 (20.3%)
Both eyes	187 (56.7%)
No	76 (23.0%)
Corneal transplantation	
Single eye	52 (15.8%)
Both eyes	18 (05.5%)
No	260 (78.8%)
Post lens implantation	
Single eye	10 (03.0%)
Both eyes	06 (01.8%)
No	314 (95.2%)
Other eye procedures	
Yes	157 (47.6%)
No	173 (52.4%)

**Figure 1:**
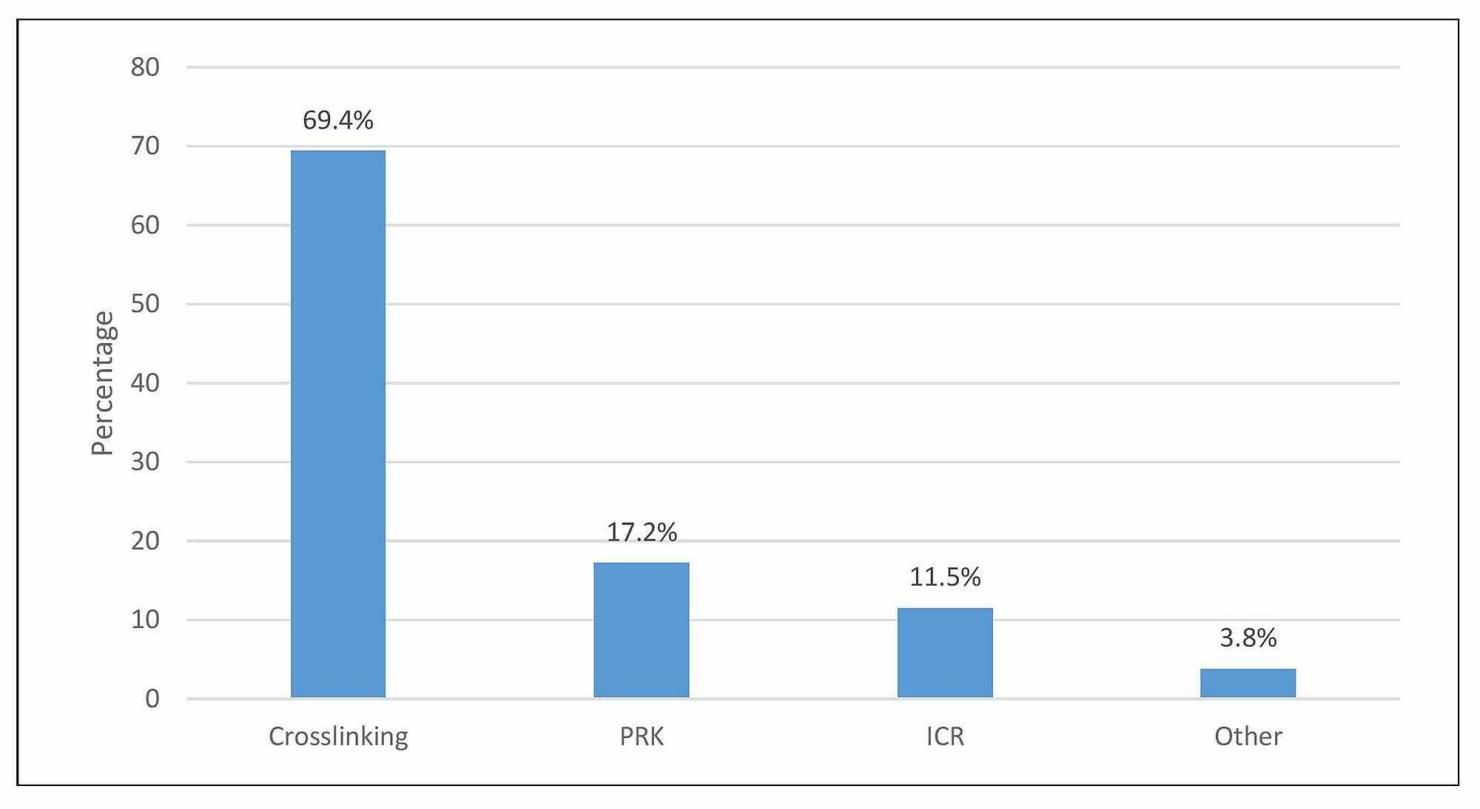
Specific other eye procedures PRK, photorefractive keratectomy; ICR, intrastromal corneal ring.

Figure 1 depicts the other eye procedures performed on the patients. It shows that crosslinking was the most common procedure performed on the patients (69.4%), followed by photorefractive keratectomy (PRK) (17.2%) and intrastromal corneal ring (ICR) (11.5%).

Table 3 shows the grades of depression. The prevalence of depression among patients with keratoconus was 40.6% while 59.4% were not depressed (mean score: 9.26 ± 5.81 SD, 95% CI: 8.26-9.89).

**Table 3:** Prevalence of depression using PHQ-9 (n = 330) SD: standard deviation, CI: confidence interval, PHQ-9: 9-item Patient Health Questionnaire.

Variables	N (%)
Total depression score (mean ± SD, 95% CI)	9.26 ± 5.81, 8.26 - 9.89
Level of depression	
Depressed	134 (40.6%)
Not depressed	196 (59.4%)
Depression severity ^(n=134)^	
Mild depression	17 (12.7%)
Moderate depression	63 (47.0%)
Moderately severe depression	36 (26.9%)
Severe depression	18 (13.4%)

**Figure 2:**
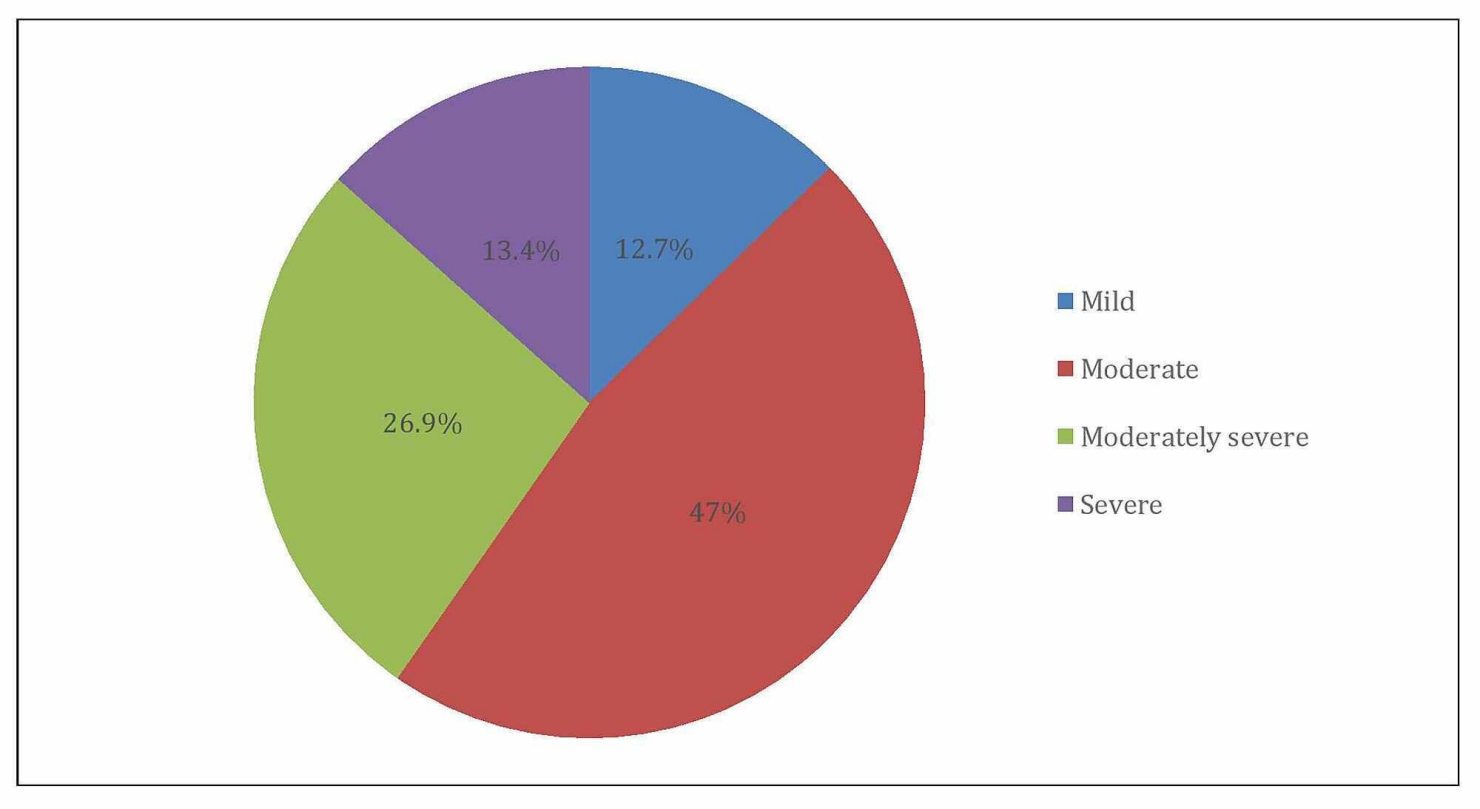
Severity of depression

When measuring the severity level, moderate depression was found among 47%, followed by moderately severe (26.9%), while severe depression was found among 13.4% (Figure 2).

When measuring the relationship between the level of depression and the socio-demographic characteristics of the patients, it was found that Saudis were significantly more depressed compared to non-Saudis (X^2^ = 7.066; p = 0.008). No other socio-demographic variables showed a significant relationship with depression other than nationality since; age group, gender, employment status, residence region and residence area did not show a significant relationship when compared to depression (all p > 0.05) (Table 4).

**Table 4:** Relationship between the level of depression and socio-demographic characteristics of patients (n = 330) §p-value has been calculated using the chi-square test. **Significant at p < 0.05 level.

Factor	Level of depression		X^2^	p-value ^§^
	Depressed N (%) ^(n=134)^	Not Depressed N (%) ^(n=196)^		
Age group				
18 – 30 years	59 (44.0%)	87 (44.0%)	0.612	0.736
31 – 40 years	62 (46.3%)	85 (43.4%)		
>40 years	13 (09.7%)	24 (12.2%)		
Gender				
Male	80 (59.7%)	116 (59.2%)	0.009	0.925
Female	54 (40.3%)	80 (40.8%)		
Nationality				
Saudi	111 (82.8%)	181 (92.3%)	7.066	0.008 **
Non-Saudi	23 (17.2%)	15 (07.7%)		
Employment status				
Unemployed	39 (29.1%)	47 (24.0%)	1.525	0.467
Employed	80 (59.7%)	130 (56.3%)		
Student	15 (11.2%)	19 (09.7%)		
Residence region				
Central region	66 (49.3%)	85 (43.4%)	2.191	0.701
Eastern region	15 (11.2%)	19 (09.7%)		
Northern region	10 (07.5%)	17 (08.7%)		
Southern region	14 (10.4%)	29 (14.8%)		
Western region	29 (21.6%)	46 (23.5%)		
Residence area				
City	111 (82.8%)	161 (82.1%)	0.026	0.871
Village	23 (17.2%)	35 (17.9%)		

When measuring the relationship between the level of depression among other eye disorders and treatment of the patients, it was found that only the use of corrective contact lens (includes both: hybrid and rigid lens) showed significant association with depression where the use of corrective contact lens in both eyes exhibited higher depression rate compared to the other groups (X^2 ^= 17.084; p < 0.001). Other variables included in the table showed no significant relationship with depression (all p > 0.05) (Table 5).

**Table 5:** Relationship between the level of depression among the patients’ other eye disorders and procedures (n = 330) *Variable with multiple responses. §p-value has been calculated using the chi-square test. **Significant at p < 0.05 level. PRK, photorefractive keratectomy; ICR, intrastromal corneal ring.

Factor	Level of depression		X^2^	p-value ^§^
	Depressed N (%) ^(n=134)^	Not Depressed N (%) ^(n=196)^		
Other eye diseases *				
Astigmatism	72 (53.7%)	88 (44.9%)	2.486	0.115
Myopia	50 (37.3%)	71 (36.2%)	0.041	0.840
Hyperopia	09 (06.7%)	12 (06.1%)	0.047	0.828
Amblyopia	10 (07.5%)	13 (06.6%)	0.085	0.771
Other	02 (01.5%)	01 (0.50%)	0.853	0.356
Use of corrective eyeglasses				
Yes	80 (59.7%)	121 (61.7%)	0.138	0.710
No	54 (40.3%)	75 (38.3%)		
Use of corrective contact lens				
Single eye	27 (20.1%)	40 (20.4%)	17.084	<0.001 **
Both eyes	91 (67.9%)	96 (49.0%)		
No	16 (11.9%)	60 (30.6%)		
Corneal transplantation				
Single eye	17 (12.7%)	35 (17.9%)	2.103	0.349
Both eyes	09 (06.7%)	09 (04.6%)		
No	108 (80.6%)	152 (77.6%)		
Post lens implantation				
Single eye	06 (04.5%)	04 (02.0%)	1.721	0.423
Both eyes	02 (01.5%)	04 (02.0%)		
No	126 (94.0%)	188 (95.9%)		
Other eye procedures				
Yes	68 (50.7%)	89 (45.4%)	0.909	0.340
No	66 (49.3%)	107 (54.6%)		
Specific other eye procedures *				
Crosslinking	47 (69.1%)	62 (69.7%)	0.005	0.941
ICR	04 (05.9%)	14 (15.7%)	3.683	0.055
PRK	15 (22.1%)	12 (13.5%)	1.991	0.158
Other	03 (04.4%)	03 (03.4%)	0.114	0.736

## Discussion

To our knowledge, this is the first study to evaluate and estimate the prevalence of depression among keratoconus patients in Saudi Arabia and the Middle East. By using PHQ-9, we found that the prevalence of depression among previously diagnosed keratoconus patients is 40.6% in various degrees of severity. This is a very high percentage considering not all participants may be having severe keratoconus. This signifies the role of keratoconus in developing depression regardless of the severity or impact on visual impairment as was found in Moschos’s study [17].

When compared to other chronic conditions, the depression prevalence among keratoconus patients in Saudi Arabia is at the middle ground between higher depression rates of sickle cell anemia (48.2%-85.9%) [22,23] and chronic pain (71%) [24] and lower depression rates of chronic kidney disease (6.8%-24.6%) [25,26] and type 2 diabetes mellitus (33.8%) [27].

Other than nationality, the socio-demographic characteristics didn’t seem to be associated with depression. Even though Saudis were significantly more depressed statistically compared to non-Saudis, we believe that the association isn’t clinically significant. A bigger sample of non-Saudis is needed for comparison to prove such an association. Further exploration of the role of ethnicity/nationality in depression among keratoconus patients is needed as this is beyond the scope of this study.

Even though depression is more common among females [11,14], we did not find a significant relationship between depression among keratoconus patients and gender. This may indicate that keratoconus is associated with depression regardless of the gender. In contrast, in Chen et al.'s study of depression among glaucoma patients; females, older patients and lower-income individuals were at a greater risk of developing depression [28].

Having a concurrent eye disease such as astigmatism, myopia, hyperopia and amblyopia isn’t significantly related to developing depression even though it has been reported that myopia and amblyopia are related to the development of depression [29,30].

The use of corrective contact lens in both eyes is associated with a significantly higher depression rate among keratoconus patients. This may be attributed to the extra amount of effort required to cope with them and their impact on daily life activities when compared to other minor daily hindrances such as corrective eyeglasses or the almost nonexistent hindrance of corneal transplantation, post-lens implantation and other eye procedures. Of course, the visual function among keratoconus patients is not identical across the different conducted eye procedures, and so it is difficult to attribute a certain eye procedure with being depressed or not. Additionally, in Saudi Arabia, the expenses of corrective contact and non-contact lens aren’t covered by the government or insurance companies which becomes a financial burden for patients with keratoconus.

A limitation of this study was not being able to exclude participants of early keratoconus with good spectacle vision as it was conducted on an online basis rather than in an ophthalmology clinic. Another limitation was the inability to measure the best-corrected distance visual acuity (CDVA). Additionally, there may be other factors associated with developing depression or having a higher depression score than usual in such patients which have not been investigated in this study, these include multiple comorbidities, grief, medications and recent traumatic events.

Further studies are needed to evaluate the factors of good spectacle vision and best CDVA on the development of depression among keratoconus patients. In addition, further exploration of the role of ethnicity/nationality in depression among keratoconus patients is needed.

## Conclusions

Depression is highly prevalent among keratoconus patients. This is especially true among corrective contact lens wearers of both eyes. Keratoconus is associated with depression regardless of disease severity and socio-demographic characteristics. We recommend screening for depression among patients diagnosed with keratoconus in ophthalmology clinics as well as primary care clinics, and to have special care provided for those using corrective contact lens in both eyes. Clinically, when treating keratoconus patients, ophthalmologists and optometrists need to provide psychological support and consider early referral to psychiatrists which could potentially improve the quality of life.
